# Partially Oxidized Sub-10 nm MnO Nanocrystals with High Activity for Water Oxidation Catalysis

**DOI:** 10.1038/srep10279

**Published:** 2015-05-22

**Authors:** Kyoungsuk Jin, Arim Chu, Jimin Park, Donghyuk Jeong, Sung Eun Jerng, Uk Sim, Hui-Yun Jeong, Chan Woo Lee, Yong-Sun Park, Ki Dong Yang, Gajendra Kumar Pradhan, Donghun Kim, Nark-Eon Sung, Sun Hee Kim, Ki Tae Nam

**Affiliations:** 1 Department of Materials Science and Engineering, Seoul National University, Seoul 151-744, Korea; 2 Research Institute of Advanced Materials (RIAM), Seoul National University, Seoul 151-744, Korea; 3 Division of Materials Science, Korea Basic Science Institute, Daejeon, 305-333, Korea; 4 Pohang Accelerator Laboratory, POSTECH, Pohang, 790-784, South Korea

## Abstract

The oxygen evolution reaction (OER) is considered a major bottleneck in the overall water electrolysis process. In this work, highly active manganese oxide nano-catalysts were synthesized via hot injection. Facile surface treatment generated Mn(III) species on monodisperse 10 nm MnO nanocrystals (NCs). Size dependency of MnO NCs on OER activity was also investigated. Surprisingly, the partially oxidized MnO NCs only required 530 mV @ 5 mA cm^−2^ under near neutral conditions.

Development of cost-effective and robust catalysts has been a demanding challenge to solving the current energy crisis^1^,^2^,^3^,^4^,^5^,^6^. Water splitting is regarded as a promising step towards environmentally sustainable energy schemes because electrolysis produces only hydrogen and oxygen, without any by-products. Within the overall water splitting process, the oxygen evolution reaction (OER), an anodic half cell reaction that generates an oxygen molecule from two water molecules, generally requires extremely high overpotential due to its slow reaction kinetics^7^,^8^,^9^,^10^. Although Ir and Ru based precious metal catalysts display moderate catalytic activity for OER, their scarcity and poor stability limit commercial application^11^,^12^,^13^. In nature, a highly efficient oxygen evolving complex (OEC) exists within photosystem II (PS II), with a core structure composed only of non-precious components, calcium and manganese (Mn_4_CaO_5_ cluster)^14^. The mixed Mn valency and presence of the redox-inactive Ca are known to be key factors for facilitating the Mn redox reaction during the OER^15^,^16^.

Inspired by the exceptional efficiency of this biological system, many research groups have attempted to mimic the OEC structure^17^,^18^,^19^,^20^,^21^. Demonstration of calcium manganese compounds such as CaMn_2_O_4_-4H_2_O, which exhibit the enhanced activity, represents a bio-inspired effort to mimic Mn_4_Ca clusters^19^. In the previous literature, Mn with mixed valency was reported to affect the Mn redox equilibria^17^. While typical Mn-oxide compounds, such as MnO_2_ and Mn_2_O_3_, have limited redox capabilities in each phase, mixed-valence Mn compounds (MnO_x_ and CaMn_2_O_4_-4H_2_O) allow for a more flexible Mn oxidation state in a single structure without severe structural deformations^17^. Moreover, Driess group synthesized amorphous MnO_x_ nanoparticles with chemical oxidation method. Active, amorphous MnO_x_ catalysts was made by oxidizing inactive, crystalline MnO nanoparticles with Ce(IV) oxidants^20^. Dau group also developed new electro-deposition method to produce MnO_x_ films which operates efficiently under neutral condition. Using XANES analysis, they found that amorphous MnO_x_ films have mixed Mn oxidation states (III/IV) and disordered Mn geometry^22^. The other active manganese oxide catalysts reported by Jaramillo group, possess Mn(III) and Mn(IV) species as well^23^.

Despite these systematic approaches, however, the main challenge associated with manganese oxide catalysis is the degraded activity under neutral conditions^24^,^25^. It has been revealed that Mn(III) is reportedly the intermediate state in the oxygen evolution reaction^26^,^27^. The d-orbital in the Mn(III) state intrinsically has a t_2g_^3^ e_g_^1^ high spin configuration, which is spontaneously subjected to a Jahn-Teller(J-T) distortion. Unless the structural flexibility is fully guaranteed, large inner stress builds up; consequently, the J-T process is inhibited but charge disproportionation (CD) occurs^28^,^29^. Therefore, this dramatic change in the manganese oxide compounds originates from the instability of the Mn(III) species during the OER process^24^,^30^.

In this study, we discovered a new Mn-based catalyst that efficiently performs water oxidation under near neutral conditions. We investigated the nanoscale effects on water oxidation catalysis and revealed a superior activity for monodisperse MnO NCs. Facile surface treatment induced the partial oxidation of Mn(II) to generate Mn(III) species, which results MnO/Mn_3_O_4_ mixed compounds. More importantly, partially oxidized MnO NCs exhibited the superior OER performance under near neutral condition, compared to previously reported Mn based catalysts.

## Results and Discussion

**Figure 1:**
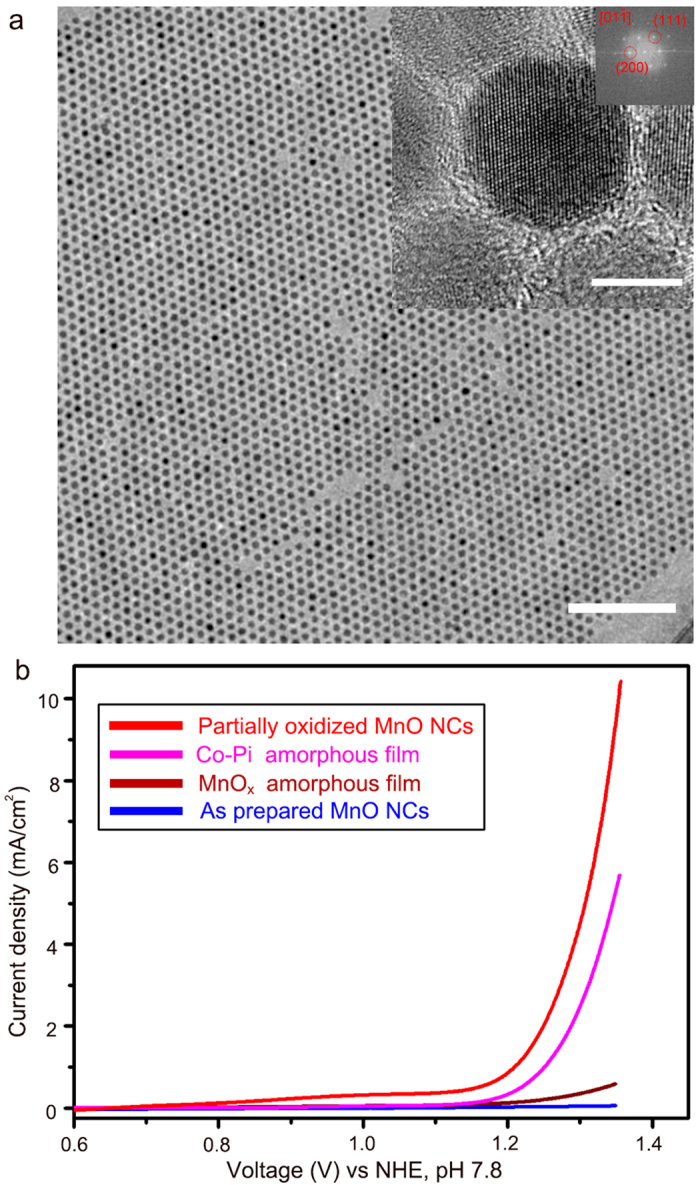
Morphology and electrochemical characterization of partially oxidized MnO NCs. **a**. HR-TEM image of the synthesized, monodisperse 10 nm MnO particles. (scale bar : 100 nm) Inset shows a high resolution image of the MnO crystal. (scale bar : 5 nm). **B**. Polarization-corrected curves for partially oxidized MnO (red), Co-Pi film (pink), MnO_x_ film (brown), and as-prepared MnO nanocrystals (blue).

Monodisperse MnO NCs were prepared via hot injection with a slight modification from previously reported literature^31^. Figures 1a and S1 show that the synthesized nanocrystals have a pure, single crystalline MnO phase and are monodisperse at sub 10 nm. High resolution TEM (HR-TEM) (Fig. 1 inset) shows the lattice fringes of the MnO (110) planes (2.54 Å) and (200) plane (2.2 Å), which matches the XRD results. (Fig. S1) Since relatively long alkyl chain is functionalized on the MnO surface during synthesis and can inhibit access of water molecusles to the MnO, eliminating the surfactant (myristic acid molecules, CH_3_(CH_2_)_12_COOH) was required to enhance the cell conductivity. To address this issue, we have conducted post surface treatment with NH_4_OH solution. To distinguish the surface-treated MnO particles from previously reported MnO particles, we refer to the 1h treated nanocrystals as “Partially oxidized MnO NCs”. XRD result indicated that partially oxidized MnO NCs have Mn_3_O_4_ /MnO mixed phases, which was also revealed with HR-TEM analysis. In addition, various electrochemical characterization such as loading amount and substrate effect were additionally investigated to reveal electrochemical properties of partially oxidized MnO NCs (Figs. S5 and S6)

The water oxidation catalytic performance of the MnO NCs was appraised via cyclic voltammetry (CV). (See the experimental section for the cell preparation details) All of the electrochemical measurements were performed at pH 7.8 in a 300 mM phosphate buffer solution. As shown in Fig. 1b, a fairly low current and high onset potential were observed for the as-prepared MnO NCs, as expected. This phenomenon was caused by the organic capping ligand around the MnO particles acting as an insulating barrier that blocked electron flow. We varied the treatment time to find out optimum condition. Figure 1b and Fig S4 showed that MnO treated with NH_4_OH for 1 h exhibited the highest catalytic performance.

We also synthesized well-known catalyst materials to quantitatively evaluate their catalytic activity. Representative amorphous metal oxide materials, Co-oxide and Mn-oxide films, were prepared via electrodeposition^10^,^32^, and their catalytic activity was compared to the partially oxidized MnO NCs. As shown in Fig. 1b, the overpotential values at a current of 5 mAcm^−2^ were 530 mV for the partially oxidized MnO NCs but 600 and 700 mV for the CoO_x_ (Co-Pi) and MnO_x_, respectively^10^,^21^,^32^.

**Figure 2:**
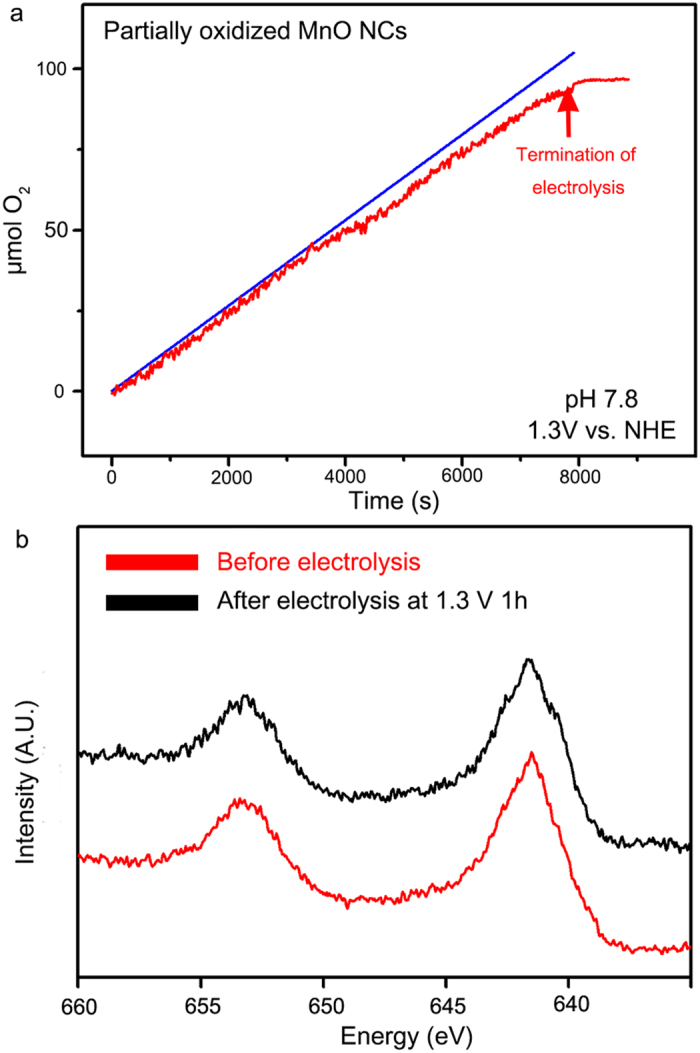
Measurment of evolved O_2_ molecules and catalytic stability test. **a**. The amount of evolved O_2_ molecules measured by experimental amount (red line) and the theoretical amount of evolved O_2_ (blue line) during bulk electrolysis. The theoretical amount of O_2_ molecules was plotted assuming a Faradaic efficiency of 100%. **b**. XPS analysis of partially oxidized MnO NCs. even after 1 h of electrolysis, there was no peak shift in Mn 2p region, which indicates the high stability of partially oxidized MnO NCs.

Faradaic efficiency experiments were performed to examine the origin of the observed current. The faradaic efficiency of partially oxidized MnO NCs was measured by a fluorescence-based O_2_ sensor. Electrolysis was performed at the applied potential of 1.3 V vs NHE. Before bulk electrolysis, the designed electrochemical cell was purged with inert gas (99.999% Ar) for 1 h, and the fluorescence sensor was located in the upper part of the cell. The trace for the amount of evolved oxygen measured by the sensor was plotted in Fig. 2a (red line). A total 95 μmol of oxygen molecules was evolved after 7900 s of electrolysis. The theoretical yield of oxygen during electrolysis was calculated from the total charge passed during the electrolysis (blue line). The faradaic efficiency of partially oxidized MnO NCs was approximately 91.95%, indicating that the current measured by cyclic voltammetry was mainly originated from the oxygen evolution reaction. Turnover frequency partially oxidized MnO NCs for at 1.18 V (η: 410 mV) was also obtained based on the electrochemical data and faradaic efficiency. Detailed calculation method was described in the supporting information materials. We would like to note that the TOF value of partially oxidized MnO NCs is calculated per Mn mol which is a lower-limit value, since the number of active sites for OER may be fewer than the number of total Mn ions in the crystals. Comparison of TOF values with other previous solid state catalysts were summarized in Table S1.

Catalytic stability of partially oxidized MnO NCs was evaluated via electrochemical analysis and *ex-situ* XPS measurement. First, we continuously cycled between the potentials 0 V and 1.3 V (vs NHE) to verify the catalytic stability. As shown in Fig. S9a, the cyclic voltammetry curves of partially oxidized MnO NCs nearly unchanged after 50 cycles, which indicates their catalytic durability. Nearly constant current was maintained during the bulk electrolysis of the partially oxidized MnO NCs under an applied potential of 1.1 V vs Ag/AgCl for 2 h. (Figure S9b) Moreover, we have examined possible change of catalyst surface. The *ex-situ* X-ray photoelectron spectroscopy (XPS) indicated the Mn(III) species on the partially oxidized MnO surfaces were stable after 1 h of bulk electrolysis at 1.3 V (Fig. 2b).

**Figure 3:**
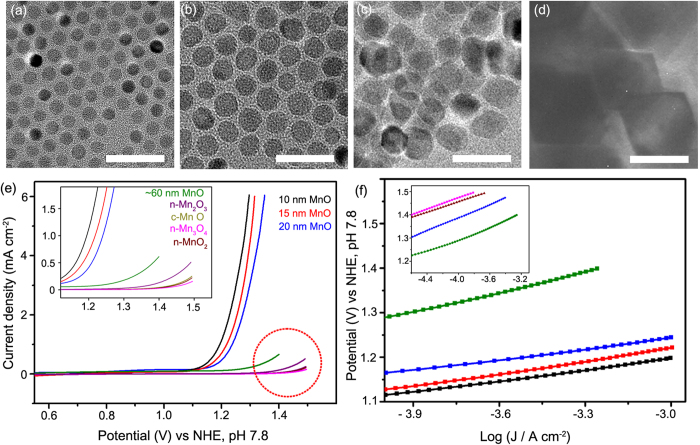
TEM images of various sized MnO NCs; **a**. 10 nm, **b**. 15 nm, **c**. 20 nm and **d**. ~60-80 nm MnO NCs. (scale bar : 50 nm) **e**. CV curves for various sized partially oxidized MnO NCs (10 nm (black), 15 nm (red), 20 nm (blue), ~60-80 nm (green)) and other conventional Mn-Oxide nano compounds (Mn_2_O_3_ (purple), commercial bulk MnO (gray), MnO_2_(brown), and Mn_3_O_4_(pink)). **f**. Tafel plots of various sized partially oxidized MnO NCs and other conventional Mn-oxide nano compounds

Various Mn-oxide nano-catalysts (MnO_2_, Mn_3_O_4_, and Mn_2_O_3_) were synthesized using previously reported methods for direct comparisons^33^,^34^,^35^. XRD and SEM analysis revealed that 70~100 nm sized Mn-oxide compounds were successfully synthesized. (Figure S7). Each Mn-oxide catalyst was mixed with a neutralized Nafion solution and loaded onto the FTO substrate via spin-coating. As expected from the previous literature^24^, the Mn-oxide catalysts displayed inferior electro-catalytic activities under neutral conditions (Fig. 3e). Additionally, we displayed electrochemical data using other metric systems with respect to the loading amount (mass activity) and surface area because each catalyst was prepared on the working electrode in a different way. The catalyst surface area on the electrode was calculated by multiplying the catalyst weight on the electrode and BET value. (Fig. S8) We have summarized the OER activity in Table 1 with respect to current density values. We concluded that the partially oxidized MnO NCs had superior activity to the previously reported Mn based OER catalysts. (Fig. S8, Table 1 and Table S1)

Tafel analysis was conducted and revealed that the exchange current density for the partially oxidized MnO NCs was much higher than for the conventional Mn-oxide nano-catalysts. The Tafel slope was ~73 mVdec^−1^ for the 10 nm sized partially oxidized MnO NCs and ~120 mVdec^−1^ for the other Mn-oxide compounds (MnO, Mn_3_O_4_, Mn_2_O_3_, and MnO_2_). (Fig. 3f)

As an effort to obtain deeper insight of the nano-size effect, we investigated the size dependency of partially oxidized MnO NCs on the water oxidation catalysis. We have prepared various sized MnO NCs and evaluated their catalytic activity. In this study, 10, 15, 20, and ~60-80 nm sized MnO NCs (Fig. 3a-d) have been synthesized via hot injection method. (See the supporting information) As shown in Fig. 3e, 10 nm particles exhibited the highest OER performance. From the Tafel analysis, almost same Tafel slope was obtained and only difference are the onset potential and exchange current values (Fig. 3f). Considering the onset potential is normally determined by intrinsic material properties, it can be inferred that a change in surface structure of the MnO particles occurred depending on their size, which affects the Mn redox reaction.

**Figure 4:**
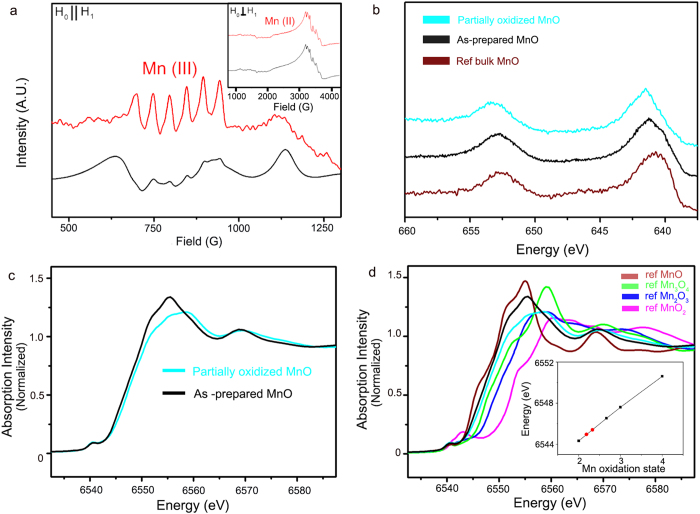
Change in the Mn oxidation state during surface treatment. **a**. Parallel mode X-band CW-EPR spectra (Inset: perpendicular mode CW-EPR spectra) and **b**. Mn 2p spectra of bulk ref MnO (brown), as prepared MnO (black) and partially oxidized MnO NCs (light blue), demonstrating Mn(II) oxidation to a higher oxidation state, Mn(III), which results in a mixed valency. (**c**, **d**) Comparison of the XANES data collected from the as-prepared (black), and partially oxidized MnO NCs (light blue) with other Mn-oxide compounds.

Electron paramagnetic resonance (EPR) and x-ray absorption spectroscopy (XAS) were conducted to identify the detailed electronic structure of the partially oxidized MnO NCs^22^,^23^,^36^,^37^. The continuous-wave EPR (CW-EPR) spectra are shown in Fig. 4a. In both the as-prepared and partially oxidized MnO NCs, a characteristic *S* = 5/2,Mn(II) signal at g ~2 with six-line^55^ Mn (*I* = 5/2, 100% abundant) hyperfine splitting was observed (Fig. 4a, inset). However, the broad EPR signal near an effective g value of ~5.7, which is characteristic of *S* = 3/2, Mn(IV), was not detected in either sample^38^. We then tried to detect another species, Mn(III), expected to be generated on the MnO NCs surface. The integer spin, known to be *S* = 2, Mn(III) is hard to observe via conventional perpendicular mode X-band EPR due to its large zero-field splitting. However, using parallel mode X-band EPR, *S *= 2, Mn(III) ions can be observed when the zero-field splitting is in an appropriate regime as observed for other manganese complexes.

Moreover, to detect rapidly decaying Mn(III) species, we prepared the EPR samples in a pyrophosphate solution, which has been shown to effectively ligate Mn(III) ions^39^. As shown in Fig. 4a, the well-resolved six-line hyperfine splitting with A ~42 G and centered at g_eff_ ~8.2 appeared in the partially oxidized MnO NCs, which indicates that Mn(III) ions were generated as speculated. In contrast, the parallel mode CW-EPR spectra of the as-prepared MnO NCs consisted of broad background signals, most likely from oxygen, with a weak Mn(III) feature. Similar results were obtained by XPS results. 10 nm sized MnO NCs exhibited more oxidized surface properties compared to reference bulk MnO compounds. Additionally, after surface treatment, positive shift was observed at Mn 2p spectra which indicated the Mn(III) generation on the surface. (Figure 4b)

The Mn K-edge spectra for each Mn compounds were recorded at room temperature, and the energy was calibrated and normalized using a glitch in the I_0_ relative to the absorption edge of Mn foil. Figure 4c shows typical Mn pre-edge features, detected near 6540 eV for all of the catalysts. A sequential shift in the XANES peak to higher energy was observed with increasing Mn oxidation state, which agrees with previous literature^22^,^23^. Corresponding with EPR and XPS results, the as-prepared MnO NCs also had a slightly higher oxidation state than bulk MnO, which was likely due to defects generated by the intrinsic nonstoichiometric nature of the Mn oxide surface. Interestingly, the partially oxidized MnO NCs exhibited higher energy than the MnO particles. Because the edge-rise energy position indicates the mean oxidation state of the manganese^22^,^23^, we could estimate the average Mn oxidation state in partially oxidized MnO NCs. As shown in the inset for Fig. 4d, the extrapolated oxidation state of the partially oxidized MnO NCs was 2.21, which was also well matched with XPS results (Fig. 4b). Combined with above described spectroscopy data, we concluded that partially oxidized MnO NCs have Mn(II/III) resting oxidation state.

**Figure 5:**
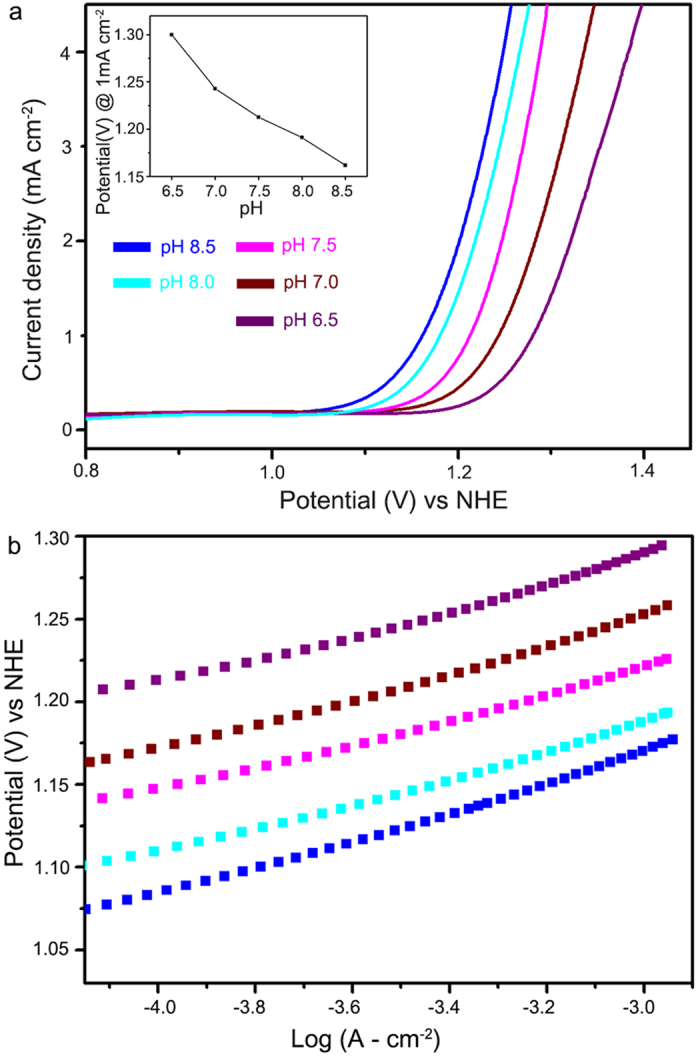
**a**. Cyclic voltammetry curves and **b**. Tafel plot of partially oxidized MnO NCs under neutral pH conditions, Inset figure indicates that partially oxidized MnO NCs follows Nernst behavior under near neutral condition.

To evaluate the detailed water oxidation mechanism of partially oxidized MnO NCs, electrochemical study was conducted. According to the previous literature and research, Tafel slope is closely related with pH dependency in anodic reaction^40^,^41^. Thus, we first evaluated pH dependency under various phosphate buffer solutions, from pH 6.5 to pH 8.5. As clearly shown in Fig. 5a, onset potential is shifted toward anodic direction as pH increased. For quantitative analysis, potential value which current density reached at 1 mA cm^−2^ was measured. Linear fit of the obtained data yields a pH dependency as 73 mV/pH (Fig. 5a inset). Secondly, we have conducted Tafel analysis and found out that under measured pH range, Tafel slope is maintained as ~70 mV dec^−1^ which almost corresponds to 2.3 RT/F. (Figure 5b)

Typically, 60~80 mV dec^−1^ Tafel slope indicates that during oxygen evolution, one-electron transfer is involved prior to rate determining step^40^,^41^. Proton activity dependence on current density could be derived as,


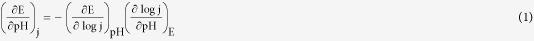


Using the obtained Tafel slope value (73 mV dec^−1^), we could know that partially oxidized MnO NCs have a first order dependency of log(j) on pH. As a result, we obtain following electrochemical raw for partially oxidized MnO NCs.


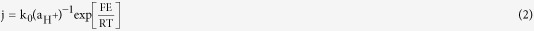


Where k_0_, a_H+_, F are potential-independent constant, proton-activity and Faraday constant, respectively. Although the whole OER catalysis process was not fully verified yet, it can be conjectured that one proton and one electron are involved prior to rate determining step in OER. Considering resting state of partially oxidized MnO NCs is Mn(II/III) and clear redox peak was observed before OER (~0.8 V), we could expect that Mn(III) → Mn(IV) is the prior step to RDS in oxygen evolving reaction.

In this study, we discovered that Mn_3_O_4_ decorated MnO NCs resulted from surface treatment, showed the superior catalytic activities compared to other Mn oxides. Overpotential to reach 5 mA cm^−2^ is just 530 mV, that is even better than widely investigated amorphous Co or Mn oxides. It has been reported that conventional Mn-oxide compounds such as Mn_3_O_4_, MnO_2_ and Mn_2_O_3_ act as inferior OER electro-catalysts, and instability of Mn(III) is considered as main reason for such a poor activity. XAS analysis and XRD analysis further proved that there was no Mn valency or phase change during OER catalysis. (Figs. S10 and S11) Thus we think that such enhancement is probably originated from the stabilization of Mn(III)^42^, in mixed Mn_3_O_4_/MnO system according to the spectroscopic analysis. Experimentally, we observed that 10 nm particles exhibited better activities than 15~20 nm particles. When the size is larger than 60 nm, the catalytic activity becomes significantly degraded to the level similar to that of bulk Mn oxides. One possible explanation that we are currently exploring is defect-rich nature and high-index facets in nano-sized MnO structure^43^.

**Figure 6:**
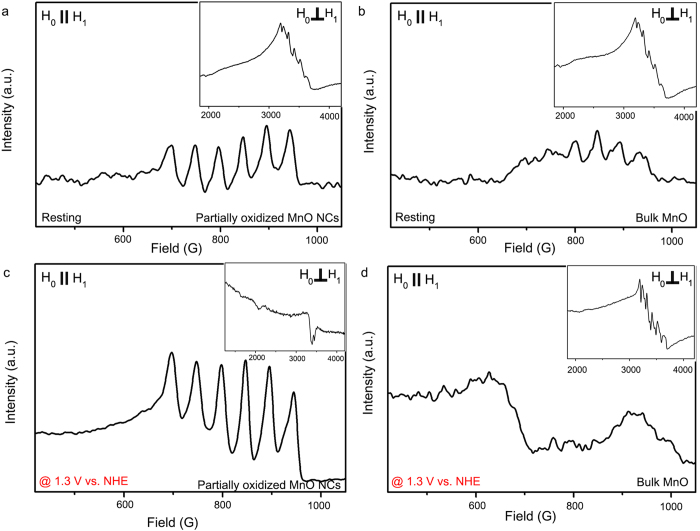
Parallel mode X-band CW-EPR spectra of partially oxidized MnO NCs in **a**. Resting state **c**. electrolysis at 1.3 V vs NHE and of reference bulk MnO compounds in **b**. resting state and **d**. electrolysis at 1.3 V vs NHE.

In this regard, we have conducted additional EPR experiments to check the stability of Mn(III) species during water oxidation catalysis. We tried to detect the Mn oxidation state during water oxidation catalysis. For comparison, we chose bulk MnO compounds. Partially oxidized nanocrystal and reference bulk MnO compounds were loaded on FTO glass (1.5 cm × 2.5 cm). The continuous-wave EPR (CW-EPR) spectra of partially oxidized MnO nanocrystals and bulk MnO compounds were displayed in Fig. 6. First, we have compared resting state of partially oxidized MnO nanocrystals with bulk MnO compounds. At resting state, bulk MnO compounds only exhibit Mn(II) signals while partially oxidized MnO nanocrystals show Mn(II) / Mn(III) signals. (Figure 6a,b) The bulk electrolysis was conducted using a cyclic voltammetry system under pH 7.8, 300 mM phosphate buffer solution. Designed potential (1.3 V vs NHE) where water oxidation occurred briskly, was applied to the sample for 30 minutes. After the bulk electrolysis, the samples were rinsed gently by deionized water and transferred to an EPR tube by blade under inert atmosphere (99.999% Ar) as promptly as possible. The EPR tube was frozen and stored at 77 K in liquid nitrogen immediately. As shown in Fig. 6c,d, EPR spectrum was altered after electrolysis was proceeded. Bulk MnO particles exhibit strong Mn(II) signals with weak Mn(III) signals and surprisingly partially oxidized MnO nanoparticles exhibit strong Mn(III) species without Mn(II) signals. This results indicate that during water oxidation reaction, Mn(III) species on partially oxidized MnO NCs are stably remained, which enable to catalyze oxygen evolving reaction efficiently. Although the exact mechanism of nano-size effect need to be further validated, our observation can suggest that nanoparticle-based approach can represent another promising direction in the development of OER catalysts.

## Conclusion

In conclusion, we reported a newly developed partially oxidized MnO nano-catalysts for the first time and evaluated its superior water oxidation catalytic activity under near neutral pH. Facile post-surface treatment created a mixed Mn valency (II/III) on the surface of MnO NCs which results high activity under near neutral condition. A size dependency was observed for the catalytic performance. Moreover, because an assembled MnO NCs works as an efficient water oxidation catalyst (Fig. S12), our system is expected to minimize the light absorption lost to the substrate, which can be essential property for photoelectrochemical (PEC) application.

## Methods

### General description for the synthesis

The manganese(II) oxide NCs were synthesized by one of the well-known thermal decomposition methods, Hot injection method. To prepare monodisperse MnO nanocrystals, first step was to make two different mixtures; one with 1 mmol of Mn(ac)_3_ and 2 mmol of myristic acid into 20 mL of octadecene and the other was to mix 3 mmol of decanol into 1 mL of octadecene. These two separate mixtures were degassed at 110 °C for 2 hours with vigorous stirring. After 2 hours of degassing, the carboxylate mixture was heated above 295 °C under argon atmosphere. When it reached to 295 °C, the mixture of decanol was injected rapidly into the carboxyl solution to induce the burst nucleation along with high super-saturation. Reaction mixture was maintained at 295 °C for 30 minutes. Various size of MnO nanoparticles could be obtained via controlling holding time at 295 °C. During the reaction, the color changes initially from clear yellow to dark brown. The dark brown solution was then cooled to room temperature and 1:1:1 ratio of the solution, acetone and toluene was mixed and centrifuged to obtain MnO precipitates. After repeating this purification step, it was re-dispersed in nonpolar solvents such as hexane or cyclohexane. For the convenience, when volume ratio of the initial dark brown MnO solution to hexane is 10, we call its concentration 1C. (For example, if we wash 100 uL of dark brown MnO solution and disperse it in 10 μL of hexane, we define concentration of final solution as 1C. Washing 200 μL of MnO solution and dispersion in 10 μL of hexane: 2C). Other reference Manganese oxide materials such as Mn_3_O_4_, Mn_2_O_3_, and MnO_2_ were synthesized using previously reported methods.

### Electrochemical Measurement

All electrochemical experiments were conducted under a three-electrode electrochemical cell system. A BASi Ag/AgCl/3M NaCl reference electrode and a Pt foil (2 cm × 2 cm × 0.1 mm, 99.997% purity, Alfa Aesar) were used as a reference electrode and a counter electrode, respectively. Electrochemical tests were carried out at ambient temperature (21 ± 1 °C) using a potentiostat system (CHI 600D, CH Instruments). Electrode potential was converted to the NHE scale, using the following equation: E(NHE) = E(Ag/AgCl) + 0.197 V. The electrolyte was phosphate buffer with 300 mM buffer strength under the pH 7.8.

### Electrochemical cell preparation

The preparation procedure of the working electrode containing our catalysts can be found as follows. First, a 30 μl of MnO solution was spin-coated onto the FTO substrate under 3000 rpm, 30 sec holding. Because its relatively long alkyl chain is functionalized on the MnO surface during synthesis and can inhibit access of water molecules to the MnO, eliminating the surfactant (myristic acid molecules, CH_3_(CH_2_)_12_COOH) was required to enhance the cell conductivity. The substrate was dipped in a 25 wt% NH_4_OH solution. Ammonia nitrogen and hydroxide ions are well-known nucleophiles and can cause nucleophilic substitution reactions. The FT-IR and XPS analysis showed that in 1h NH_4_OH treated MnO NCs, the peaks typical for myristic acid were sigificantly removed, and almost disappeared after 3 h treatment. (Figure S2). As shown in Figure S3, after post heat treatment, similar activity was observed.In addition, as shown in Fig. S12, the partially oxidized MnO NCs were still well assembled on the FTO substrate. Finally, ammonia treated FTO substrate was rinsed with deionized water and annealed at 150 °C 1h. Prior to every electrochemical experiment, the solution resistance was measured in the electrolysis bath. All the data were iR-compensated.

### EPR Study

All EPR measurements were carried out at KBSI, Daejeon, Korea. Electron paramagnetic resonance (EPR) was performed using a Bruker EMX/Plus spectrometer equipped with a dual mode cavity (ER 4116DM). Low temperatures were achieved and controlled using a liquid He quartz cryostat (Oxford Instruments ESR900) with a temperature and gas flow controller (Oxford Instruments ITC503). The experimental conditions are as follows. Microwave frequency 9.64 GHz (perpendicular mode), 9.4 GHz (parallel mode), modulation amplitude 10 G, modulation frequency 100 kHz microwave power 0.94 mW (perpendicular mode), 5.0 mW (parallel mode) temperature 5.7 K. 10 scans were added for each spectrum. All the samples were loaded on FTO glass (1.5 cm × 2.5 cm). To eliminate residual Mn(III) ion on the surface of monodisperse MnO nanoparticles, the paste-loaded FTO glass was dipped in 20 mM pyrophosphate solution at least 30 minutes and gently rinsed with deionized water prior to analysis.the samples were transferred to an EPR tube by blade under Ar (99.999%) atmosphere as promptly as possible. The EPR tube was frozen and stored at 77 K in liquid nitrogen immediately.

### XRD, SEM, TEM, BET, and XPS analysis

Powder X-ray diffraction (XRD) was carried out on a D-8 Advance X-ray diffractometer with Cu Kα radiation (λ = 1.54056 Å). For the measurement, washed MnO nano powder was collected and lyophilized at least 2 days. The lyophilized powder was loaded on Si holder, retrofitted in X-ray diffractometer. XRD patterns were recorded in a range of 5~60° with a step of 0.02° and a velocity of 0.02°/4 s. Obtained XRD patterns were compared with previously reported JDPDS cards. The morphology of synthesized MnO on the FTO glass was characterized with a high resolution scanning electron microscope (Supra 55VP, Carl Zeiss, Germany). After deposition of MnO, the substrate was rinsed gently with deionized water at least 3 times and dried with nitrogen gas. Images were taken with an acceleration voltage of 2 kV, and EDX spectra with a 15 kV. Transmission electron microscopy (TEM) images and selected area electron diffraction (SAED) patterns were obtained using a high resolution transmission electron microscope (JEM-3000F, JEOL, Japan) with an acceleration voltage of 300 kV. For the analysis, dispered MnO solution was dropped on the TEM grid and dried in an oven. For the stability test, the TEM samples were collected from FTO glass right after electrochemical measurement, and dispersed in ethanol by sonication about 1 min. About 10 μl of dispersed partially oxidized MnO NCs were dropped on the TEM grid and dried in an oven. Brunauer-Emmett-Teller (BET) analysis was conducted on the lyophilized monodisperse MnO nano powders and reference Mn-oxide nanocatalysts. Each sample was loaded to BET analyzer (Physisorption Analyzer, micromeritics, USA) under N_2_ adsorption environment. X-ray photo-electron spectroscopy (XPS) spectra were obtained by electronspectroscopy (Sigma Probe; Thermo VG Scientific, UK) with a pass energy of 30 eV and a step size of 0.1 eV. All the binding energies are referenced to C 1s (284.5 eV).

## Author Contributions

K.J, A.C, D.J, and S.E.J synthesized and characterized the materials. K. J, J. P, D.J, U.S, H.-Y.J, C.W.L, K.D.Y, Y.-S.P, and G.K.P carried out electrochemical measurements and analyzed the data. K.J, D.K and S.H.K performed EPR measurements. K.J, J. P, D.J, S.E.J, and N.-E.S carried out XAS experiments. K.J and K.T.N wrote the manuscript and prepared the figures. K.T.N directed the research. All authors reviewed the manuscript.

## Additional Information

**How to cite this article**: Jin, K. *et al*. Partially Oxidized Sub-10 nm MnO Nanocrystals with High Activity for Water Oxidation Catalysis. *Sci. Rep.* **5**, 10279; doi: 10.1038/srep10279 (2015).

## Supplementary Material

Supplementary Information

## Figures and Tables

**Table 1:** Comparison of OER activity for various water oxidation catalysts.

**OER Catalyst**	**Preparation condition**	**Overpotential at Neutral pH**	**Notes**
**Partially oxidized MnO NCs**	Spin coating	**530 mV (@ 5 mAcm**^**−2**^)	**This work**
Co-Pi	Electro-deposition	570** **mV (@ 5 mAcm^−2^)	Reproduced with the previous method (ref 10)
MnO_x_	Electro-deposition	600** **mV (@ 1 mAcm^−2^)	Reproduced with the previous method (ref 25)
Mn_3_O_4_	Spin coating	650** **mV (@ 40 μAcm^−2^)	Fails to reach 5 mA/cm^2^
Mn_2_O_3_	Spin coating	490** **mV (@ 40 μAcm^−2^)	Fails to reach 5 mA/cm^2^
MnO_2_	Spin coating	630** **mV (@ 40 μAcm^−2^)	Fails to reach 5 mA/cm^2^
IrO_x_	Electro-deposition	310** **mV (@ 5 mAcm^−2^)	Extrapolation from ref 12
