# Supplementary material for: Partially Oxidized Sub-10 nm MnO Nanocrystals with High Activity for Water Oxidation Catalysis
Source: Sci Rep. 2015 May 22;5:10279. doi: 10.1038/srep10279 (PMC4441116; doi:10.1038/srep10279)
Supplement: Supplementary Information [file srep10279-s1.pdf]

Supporting Information

Partially Oxidized Sub-10 nm MnO Nanocrystals with High Activity for Water Oxidation Catalysis

Kyoungsuk Jin[†,‡], Arim Chu[†,‡], Jimin Park[†,‡], Donghyuk Jeong[†,‡], Sung Eun Jerng[†,‡], Uk Sim[†,‡], Hui-Yun Jeong[†,‡], Chan Woo Lee[†,‡], Yong-Sun Park[†,‡], Ki Dong Yang[†,‡], Gajendra Kumar Pradhan[†,‡], Donghun Kim[#], Nark-Eon Sung[§], Sun Hee Kim[#], and Ki Tae Nam[†,‡,]*

A. Materials

$Mn(CH_3COO)_3$-$2H_2O$ (99 %), 1-Octadecene (90%), Myristic acid (99%), Decanol ($CH_3(CH_2)_9OH$), Na_2HPO_4-$7H_2O$ (ACS reagent, 98.0-102.0 %), and NaH_2PO_4-$2H_2O$ (99.0 %) were purchased from Sigma Aldrich and used as received without further purification. Fluorine doped Tin Oxide coated glass (FTO, TEC-8) which has 15 Ω sq^{-1} surface resistivity was obtained as pre-cut by 1.0 cm × 1.5 cm glass pieces from Pilington Company.

B. Supporting figures and Tables (Figure S1~S11 & Table S1)

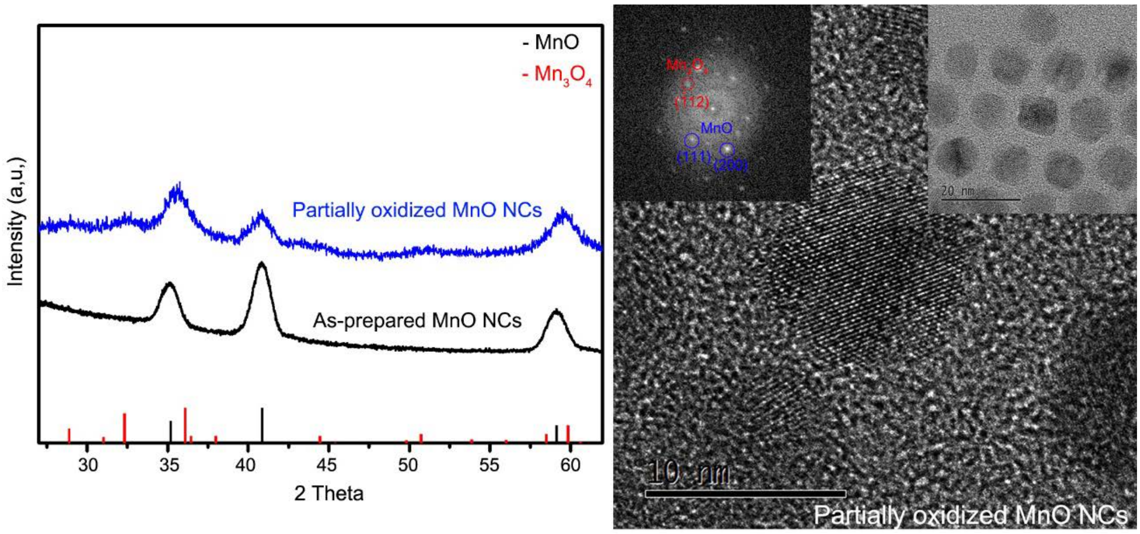

Figure S1 X-ray diffraction results of as-prepared and partially oxidized MnO nano crystals. As clearly shown in the Figure S1b, partially oxidized MnO NCs have MnO and Mn_3O_4 mixture phases.

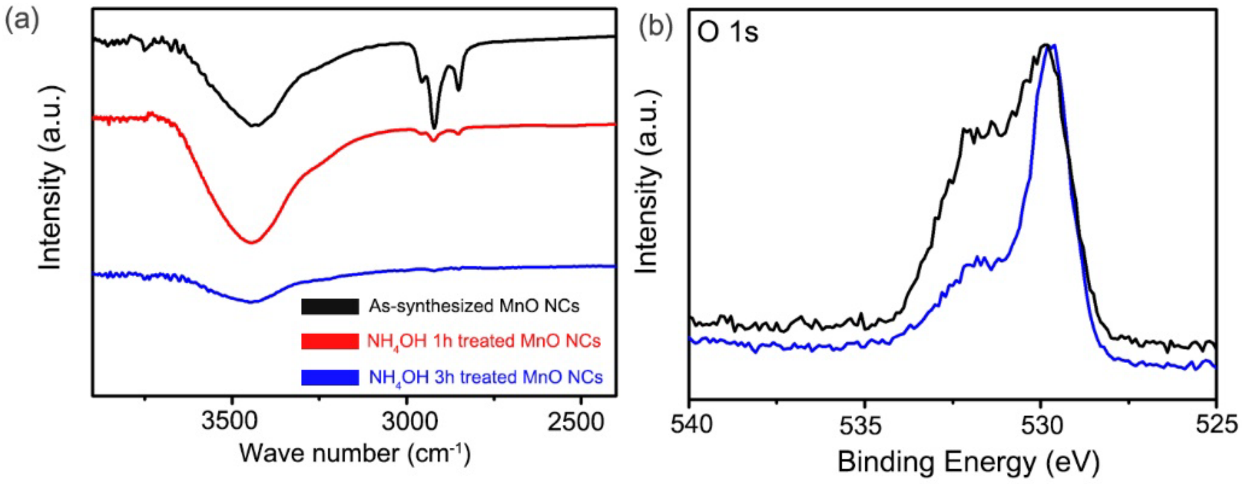

Figure S2 Fourier transform infrared (FT-IR) spectra of as-prepared, and partially oxidized MnO NCs. As shown in the figure, characteristic peak of alkyl chain around 2800 cm^{-1} was almost disappeared after surface treatment. This result indicates that long organic chain on the synthesized MnO surface was successfully eliminated by ammonia treatment.

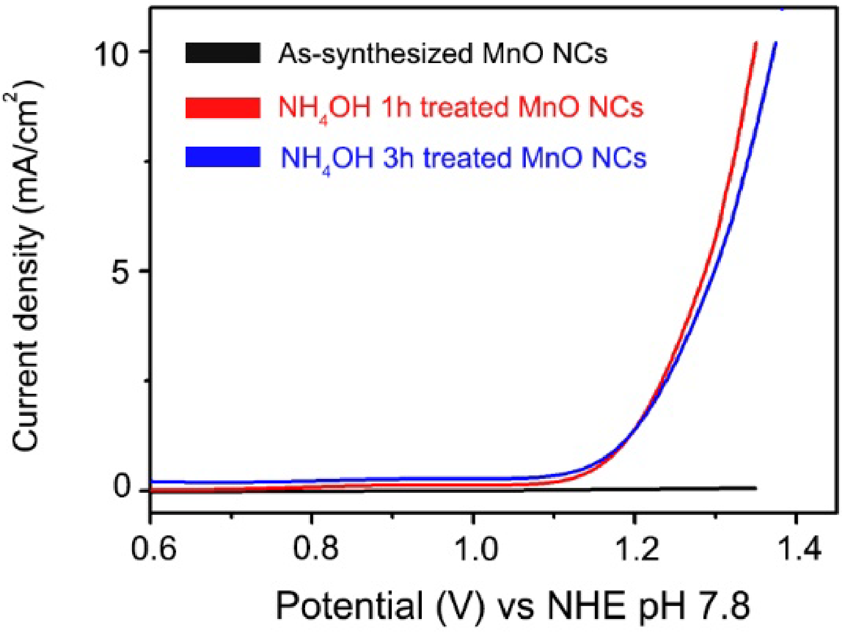

Figure S3 CV curves of Partially oxidized MnO NCs depending on NH_4OH treatment time. As shown in Figure S3, after post heat treatment, similar activity was observed for both condition.

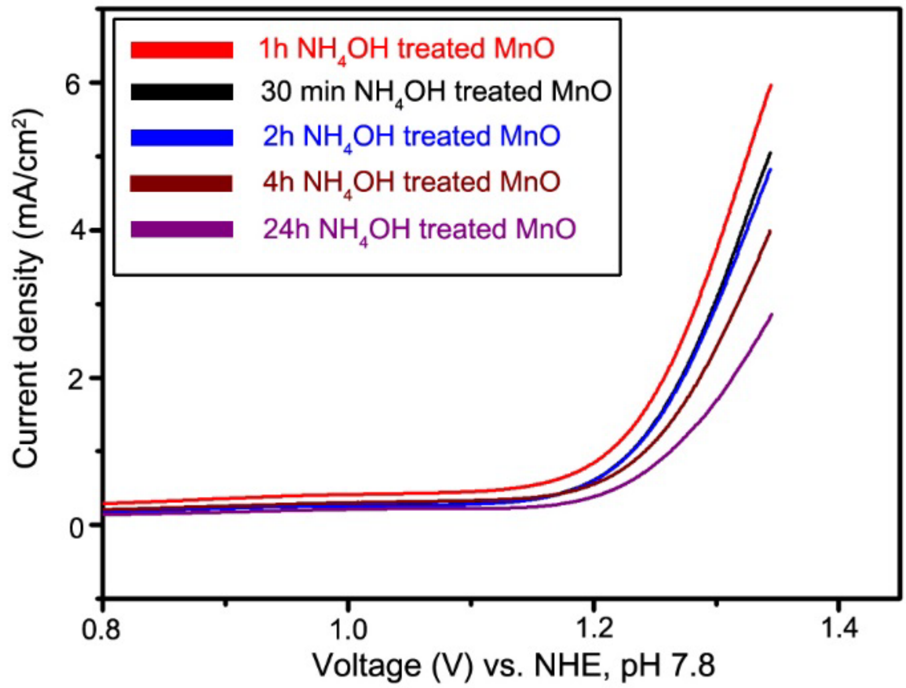

Figure S4 Cyclic voltammetry curves of drop-casted MnO nanoparticles before annealing. As shown in the Figure S4, 1h NH_4OH treated MnO nanoparticles exhibit the highest OER catalytic performance.

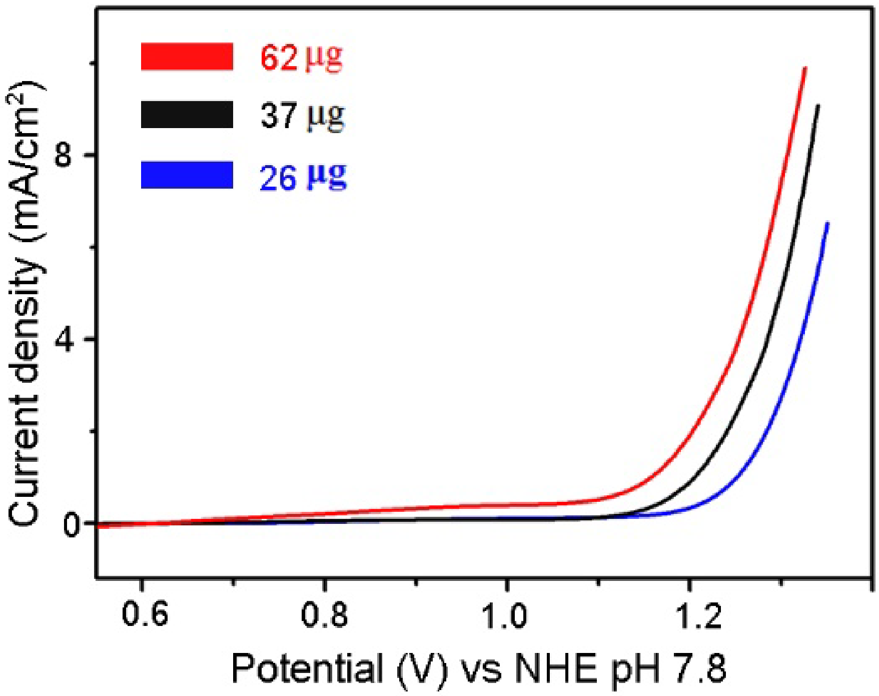

Figure S5 Dependency of the water oxidation activity on loading amount of MnO nanoparticles. As shown in Figure S5, when the loading amount increased, catalytic current were also enhanced. We think that catalytic performance could be improved due to the increase of active site for water oxidation.

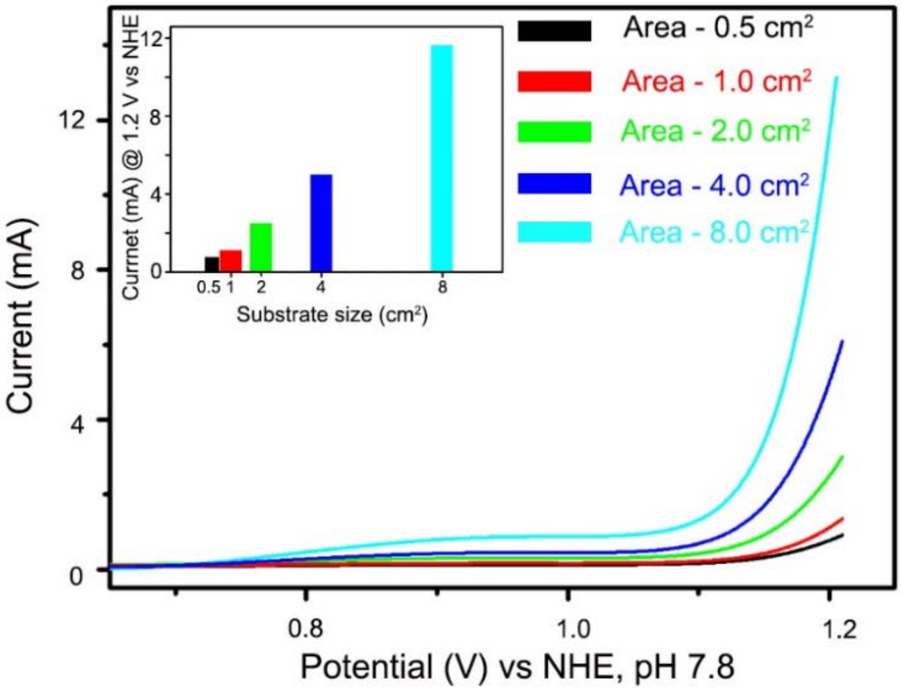

Figure S6 Cyclic voltammetry curves of partially oxidized MnO NCs prepared on various FTO substrate size As clearly shown in the Figure, OER current linearly are enhanced as the size of substrate increased.

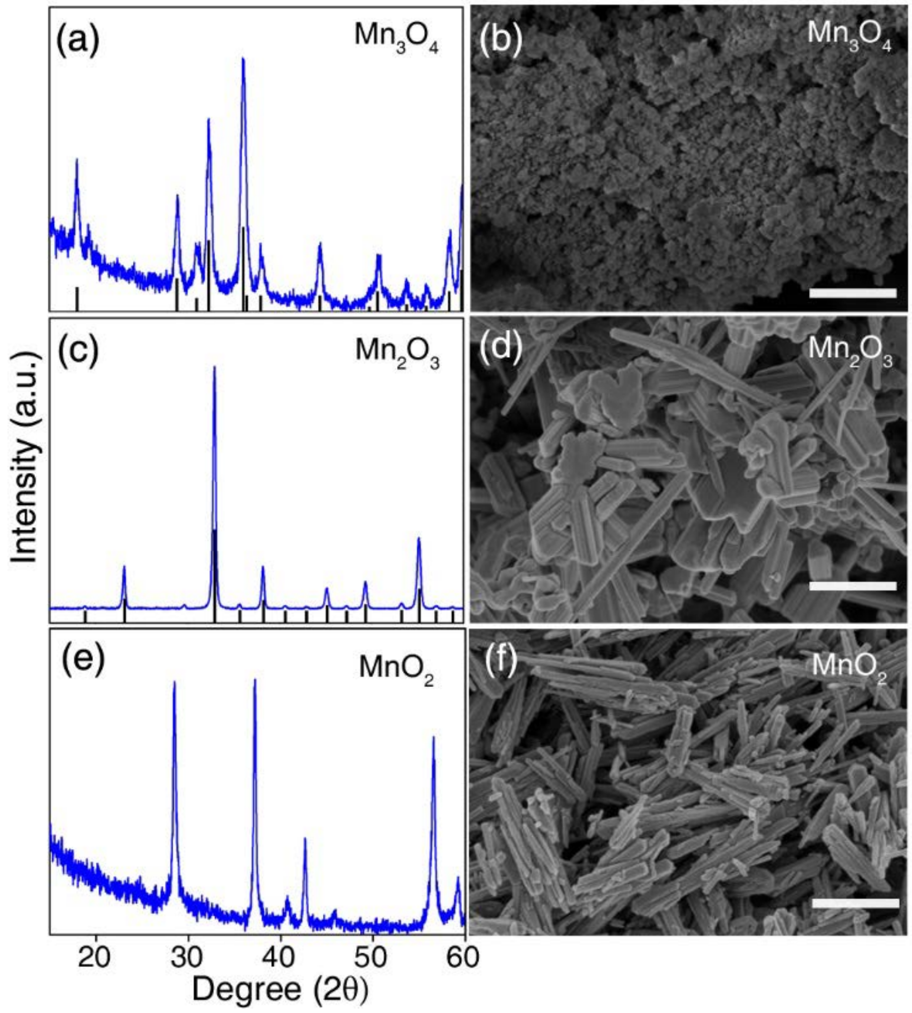

Figure S7 Characterization of synthesized Mn_3O_4, Mn_2O_3 and MnO_2 nanoparticles. (a, c, and e) XRD patterns and (b, d and f) SEM images of, Mn_3O_4, Mn_2O_3 and MnO_2 nanoparticles. (Scale bar : 600 nm) The XRD analysis clearly showed that all of the synthesized Mn-oxide nanoparticles had monophasic features without containing a secondary phase. Mn_3O_4 nanoparticles were spheres that were 50~100 nm in size, while Mn_2O_3 and MnO_2 were nanorods that were 80~300 nm in size.

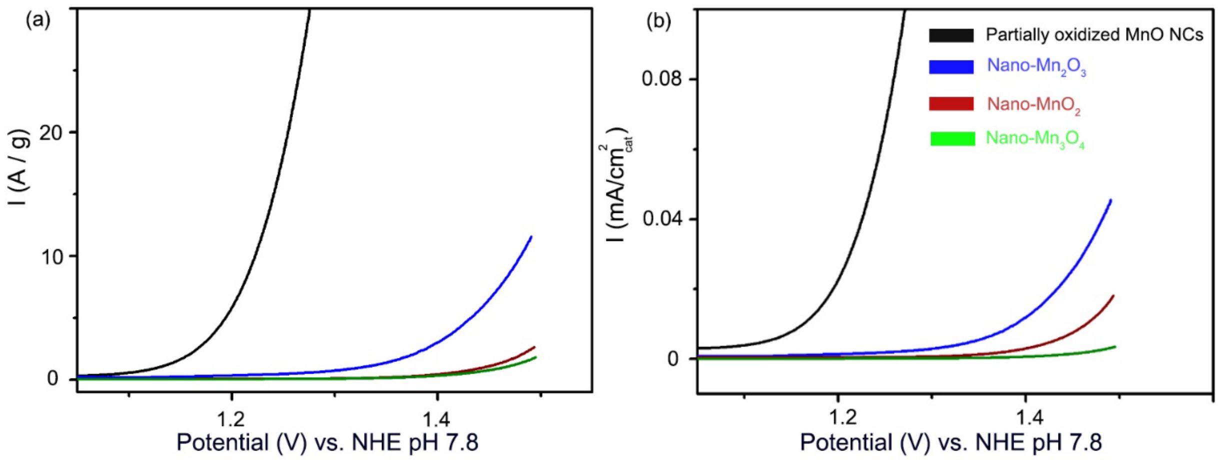

Figure S8 Polarization corrected curves of partially oxidized MnO NCs (black) and conventional Mn-oxide nano-compounds, Mn_2O_3 (blue), MnO_2 (brown), and Mn_3O_4 (green). Catalytic current value is normalized based on the (a) loading amount and (b) surface area of each catalysts. Surface area of each catalysts was obtained by BET analysis. (partially oxidized MnO NCs: 28.08 m^2/g, nano Mn_2O_3 : 25.43 m^2/g, nano Mn_3O_4: 52.76 m^2/g and MnO_2 : 14.61 m^2/g)

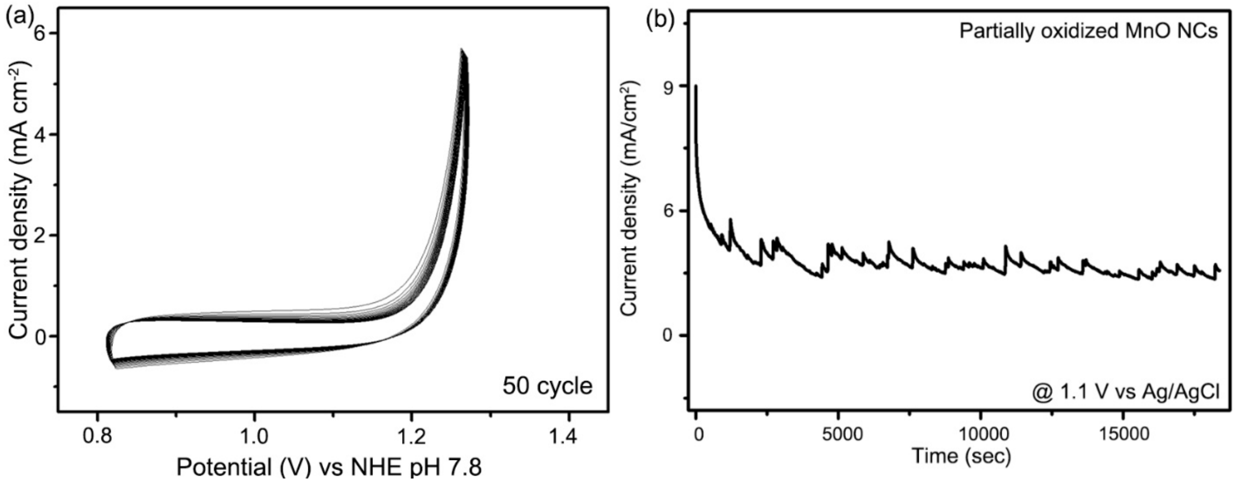

Figure S9 OER polarization curves of partially oxidized MnO NCs on FTO under 50 cycles of accelerated stability test. (d) Chronoamperometry analysis of partially oxidized MnO NCs.

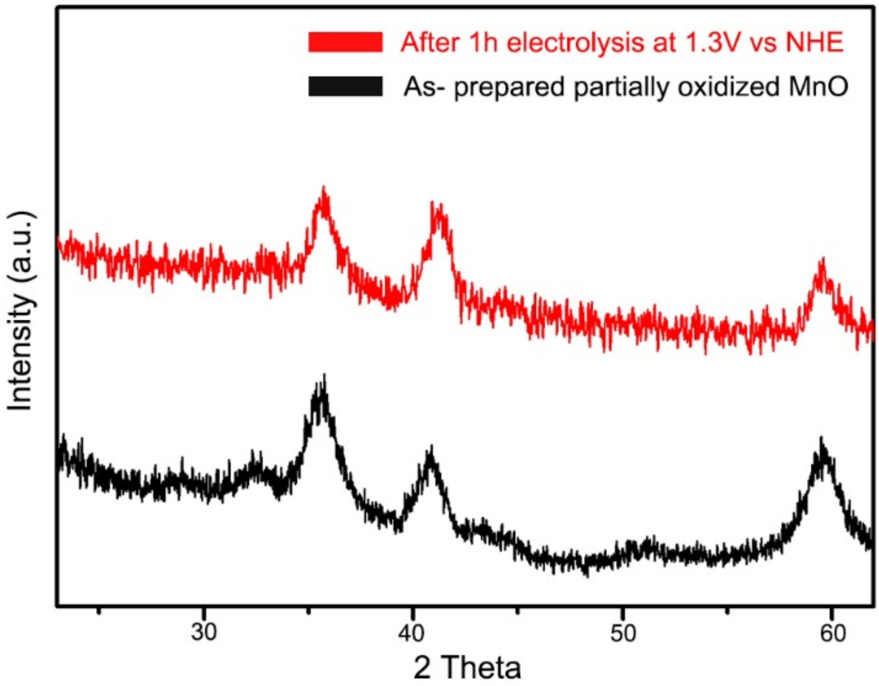

Figure S10 Comparison of X-ray diffraction pattern between as-prepared partially oxidized MnO NCs (black) and after 1h electrolysis(red) at 1.3V vs NHE (red), which indicates the phase stability of partially oxidized MnO NCs.

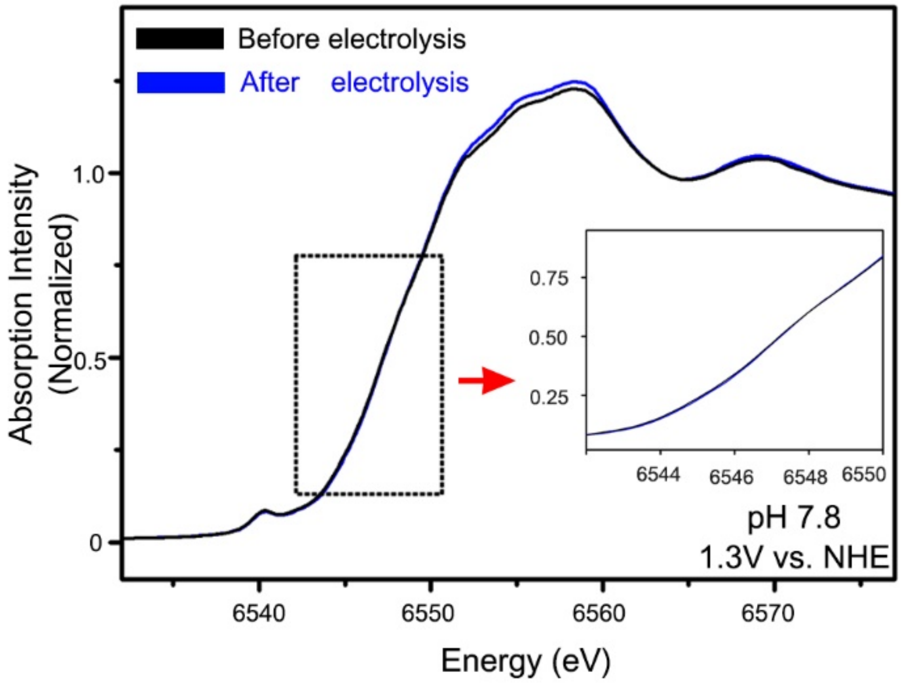

Figure S11 XANES spectra corresponding to the Mn K-edge of partially oxidized MnO NCs. The electrochemical cell for XANES analysis was designed as reported in the previous reports.[38] Partially oxidized MnO NCs was electrolyzed at the applied potential of 1.3 V vs NHE for 1 h. The XANES spectra corresponding to the Mn K-edge spectra of partially oxidized MnO NCs were recorded. As shown in Figure S11, there was no peak shift between before and after bulk-electrolysis at an applied potential of 1.3 V vs NHE for 1 h.

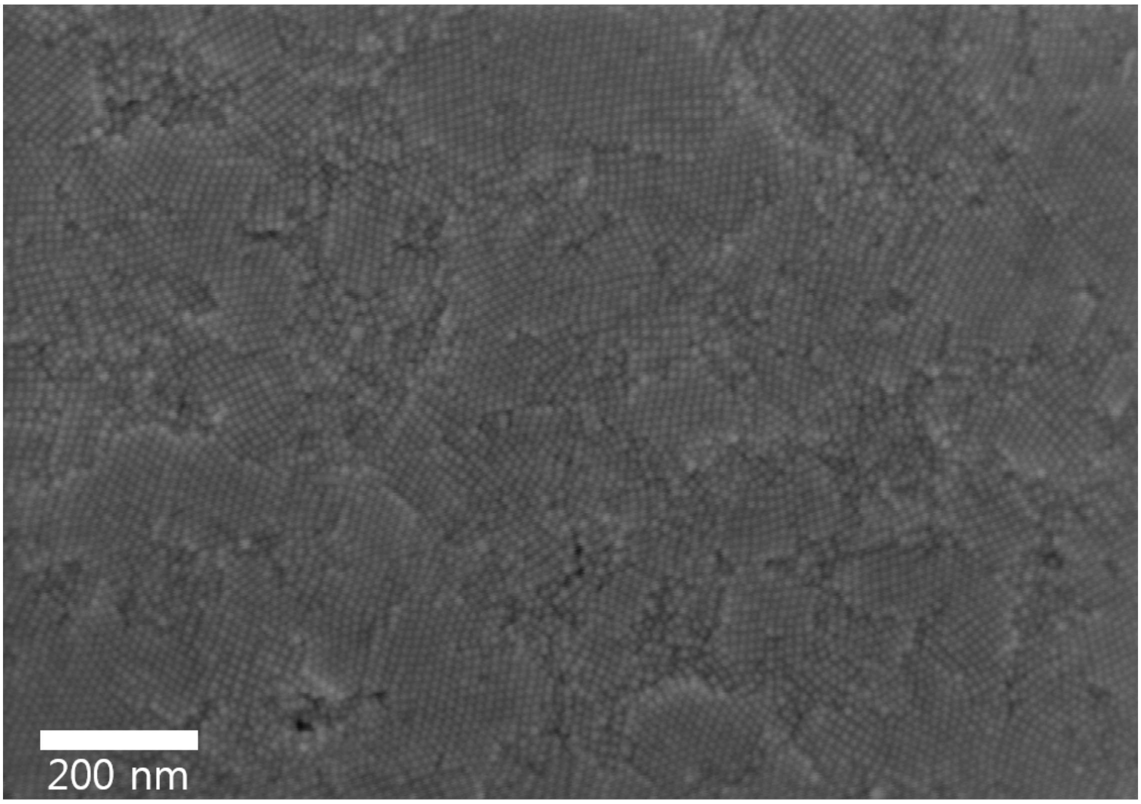

Figure S12 SEM images of MnO nanoparticles on the FTO substrate. Sub 10 nm sized MnO nanoparitcles are well assembled after deposition on the FTO substrate.

Catalyst	TOF (s^{-1})	Experimental Condition
**Partially oxidized MnO NCs[S1]**	**0.012**	***Electrochemical*** **pH 7.8 (η : 410mV)**
Co-Pi[S2]	>0.0007	*Electrochemical* pH 7 (η : 410mV)
**α**- MnO_2 nanowire[S3]	$5.9*10^{-4}$	*Photochemical* $Ru(bpy)_3Cl_2$ - $Na_2S_2O_8$,No buffer, pH 7
CO_3O_4[S4]	> 0.0025	*Photochemical* Na_2SO_4 electrolyte, pH 7 (η : 350mV)
Mn_2O_3[S4]	0.055	*Photochemical* KOH electrolyte (η : 325mV)
MnO_2[S5]	0.013	*Electrochemical* Phosphate buffer, pH 7 (η : 440mV)
IrO_2[S6]	7	*Electrochemical* pH 5.3 (η : 570mV)
Ru-red/Pt-black[S6]	0.417	*Electrochemical* pH 5.3 (η : 570mV)

Table S1 The TOF values of the various manganese oxide catalysts that have been reported by others and of our catalyst, partially oxidized MnO NCs. The detailed experimental conditions for photo and electrochemical catalysis are summarized in the third column. Turnover frequency (TOF) value in this work is estimated from the following equation.

$$\mathrm{TOF} = \frac{\mathrm{J} \times \mathrm{A} \times \eta}{4 \times \mathrm{F} \times \mathrm{m}}$$

J (A-cm^{-2}) is the measured current density. A is the area of the FTO substrate. η is faraday efficiency. F is the faraday constant and m is the number of moles for the active catalysts, prepared on the FTO substrate.

Reference

[S1] : *This work*
[S2] : *Science* **321,** 1072 (2008)
[S3] : *Chem. Comm.* ***47,*** 8973 (2011)
[S4] : *J. Chem. Soc. Faraday Trans.1* **84,** 2795 (1988)
[S5] : *Electrochim. Acta* **22,** 325 (1977)
[S6] : *J.Phys. Chem. B.* **109,** 21489 (2005)
